# PhyloExplorer: a web server to validate, explore and query phylogenetic trees

**DOI:** 10.1186/1471-2148-9-108

**Published:** 2009-05-18

**Authors:** Vincent Ranwez, Nicolas Clairon, Frédéric Delsuc, Saeed Pourali, Nicolas Auberval, Sorel Diser, Vincent Berry

**Affiliations:** 1 Institut des Sciences de l'Evolution (ISEM, UMR 5554 CNRS), Université Montpellier II, Place E. Bataillon – 34095 Montpellier Cedex 05, France; 2 Equipe Méthodes et Algorithmes pour la Bioinformatique, LIRMM (UMR 5506 CNRS), Université Montpellier II, Place E Bataillon – 34095 Montpellier, France

## Abstract

**Background:**

Many important problems in evolutionary biology require molecular phylogenies to be reconstructed. Phylogenetic trees must then be manipulated for subsequent inclusion in publications or analyses such as supertree inference and tree comparisons. However, no tool is currently available to facilitate the management of tree collections providing, for instance: standardisation of taxon names among trees with respect to a reference taxonomy; selection of relevant subsets of trees or sub-trees according to a taxonomic query; or simply computation of descriptive statistics on the collection. Moreover, although several databases of phylogenetic trees exist, there is currently no easy way to find trees that are both relevant and complementary to a given collection of trees.

**Results:**

We propose a tool to facilitate assessment and management of phylogenetic tree collections. Given an input collection of rooted trees, PhyloExplorer provides facilities for obtaining statistics describing the collection, correcting invalid taxon names, extracting taxonomically relevant parts of the collection using a dedicated query language, and identifying related trees in the TreeBASE database.

**Conclusion:**

PhyloExplorer is a simple and interactive website implemented through underlying Python libraries and MySQL databases. It is available at:  and the source code can be downloaded from: .

## Background

### Motivation

Evolutionary biologists now have to deal to an increasing extent with many phylogenetic trees and user-friendly bioinformatic tools are needed for handling large tree collections. Such tools are especially relevant for tasks that are cumbersome to perform manually, such as validating taxon names using a reference taxonomy, providing taxon sampling statistics, or pruning source trees so that they only contain taxa from a taxonomic group of interest. Such questions are crucial in phylogenomics, where phylogenies are mainly obtained by concatenating gene sequences into large molecular datasets (e.g. supermatrix approach) or by combining individual trees inferred separately on each gene (e.g. supertree approach). Several problems addressed here from a tree framework standpoint only take their constitutive taxa into account. Described solutions are thus also suitable for many other kinds of data (e.g. sequence alignments, morphological measures), for which taxonomic representation is essential.

### Problems to solve

Knowing how many different taxa belonging to a particular taxonomic group (e.g. placentals) are represented in a tree collection, determining how many trees contain at least one placental, finding these trees, and pruning them from every taxon except mammals are various tasks that cannot be performed without mapping user taxa to a reference taxonomy. It is relatively complicated to compute such a mapping [1], but the results can enable powerful tree search, while also providing pruning functions and relevant statistics.

#### Dealing with taxon names

Because of the multiplicity of phylogeny origins and the lack of a single reference taxonomic framework, some taxon names they host are often taxonomically invalid, misspelled, and/or supplemented with indications relative to primary data such as sequence accession numbers, geographical origins of the samples, marker identifiers, etc. An initial step has thus to be performed to standardize names so that software analyzing the collection will not consider them as different taxa. Besides, mapping to proper scientific names may fail for some taxon names. This problem has already been pointed out [1] and was recently addressed for the TreeBASE repository [2]. Indeed, TreeBASE is a very helpful collaborative resource but it has been largely under-exploited, partly due to its lack of taxonomic consistency [3].

#### Obtaining statistics on taxon coverage in a tree collection

As the number of trees and considered taxa grows, statistics become crucial for depicting the collection content, and thus measuring its relevance for a given phylogenetic problem. In a phylogenomic context, having hundreds of genes for dozens of taxa gives rise to simple, but fundamental questions, such as: How sparse is the dataset? Is the taxonomic sampling homogeneous among the main taxonomic groups? How complete is the taxonomic coverage of the dataset? How many genes provide information for a taxon that appeared in an unexpected position in the final phylogeny? The frequency of appearance of each taxon, although useful, is less informative than a trees × taxa presence matrix. Such a data availability matrix [4] clearly highlights under-represented taxa and provides an intuitive picture of the dataset sparseness. However, it is not sufficient to grasp the taxonomic coverage of a given group.

#### Finding trees with taxa of interest

With a large collection of trees at hand, a key task is to find those containing a relevant taxon sample with respect to a targeted biological question. Indeed, as the number of phylogenomic projects increases, it is becoming easier to access large datasets used in previous phylogenomic studies or stored in dedicated repositories such as TreeBASE [2], Homolens [5], TreeFAM [6], OrthoMam [7], and EnsEMBL [8]. For example, when studying the phylogeny of rodents, one can be interested in isolating trees with at least four rodents from TreeBASE or from databases developed from larger-scale phylogenomic analyses (e.g. focused on mammals or eutheria). A tool for exploring tree collections should therefore incorporate a feature allowing complex queries on trees according to taxonomy.

#### Pruning trees according to taxa of interest

Even trees containing several relevant taxa may contain numerous additional taxa that are deemed irrelevant for subsequent analyses. These useless parts should be pruned from trees both to speed the forthcoming analyses and to avoid interference with the signal of the core data to be analyzed. It is thus useful to have a tool allowing automatic pruning of numerous source trees so that they will only contain taxa belonging to the taxonomic groups of interest.

## Implementation

### Taxon naming convention

In order to manage tree collections, taxon names first have to be identified and then placed in a reference taxonomic scheme. PhyloExplorer allows the user to choose between two such schemes: the NCBI Taxonomy [9] and the Catalogue of Life 2008 Annual Checklist [10] from the Integrated Taxonomic Information System (ITIS) [11]. Most published phylogenies use a liberal naming scheme that combines taxonomic information with other kinds of information such as gene names, sequence identifiers or geographical origins. For example, when a tree inferred using the BRCA1 gene contains a domestic mouse among its taxa, its label can be either: mouse, BRCA1_mouse, Musmusculus_NM_015745, and so on. This freedom may allow the user to uncover some relevant information, but it also impedes simple automatic determination of the taxon represented by the given name. We use a naming convention to facilitate this determination without any loss of generality. The principle is to use separator characters chosen by the user to distinguish among the various pieces of information encompassed in the taxon name. The complete name is thus split into several words, with the first ones being used to identify this taxon within the reference taxonomy while others can be used freely for storing any other kind of information. We adopted this naming convention because it is so frequently used in TreeBASE that it almost seems to be a *de facto *standard. Most taxonomic terms consist of a single word (e.g. higher-level taxa), but some consist of two distinct terms to reflect the Linnean "genus-species" classification or to avoid ambiguity. Indeed, the reference taxonomy may contain ambiguous names because of name homonymy and synonymy. For example, *Echinops *is both a plant genus and a mammal genus. In this case, two distinct taxa have the same genus name and additional information can be provided between "<" and ">" to overcome this ambiguity. In the NCBI taxonomy, *Echinops *is annotated as either "*Echinops *< mammal >" or "*Echinops *< plant >". When a taxon name is composed of several words, PhyloExplorer first checks if the whole name corresponds to a term of the reference taxonomy. If the check fails, the last word is ignored and the thus-obtained shorter name is checked against the taxonomy – this shortening operation is repeated until the name is found within the taxonomy or is reduced to an empty string. For example, the following names respect our naming convention: mus, mus_BRCA1, mus_musculus_BRCA1_France, echinops_<mammal>_BRCA1, when using the underscore character as a separator.

### Mapping TreeBASE

PhyloExplorer also proposes a function that allows users to perform taxonomic queries on trees stored in TreeBASE [2]. For this purpose, the content of TreeBASE was obtained from its ftp server . The 3,696 studies available as of September 2008, containing a total of 6,237 trees and 93,013 taxa, were collected. Since the trees available in TreeBASE have been provided by different scientists without adopting a common naming convention, a considerable proportion of the taxon names are not scientific names present in the reference taxonomies. For TreeBASE taxon names not present in the NCBI taxonomy (i.e. less than 33%), we relied on the TBMap tables which translate most TreeBASE taxa into proper scientific names based on a number of taxonomic databases (IPNI, uBio, ITIS, RDMP) [3]. This allowed identification of a further 8% of the taxon names.

### PhyloExplorer implementation overview

PhyloExplorer is written in the Python language and has been implemented according to the Django web framework [12]. Django is a stable and powerful system that is able to handle thousands of page views per second and to associate a graphical user interface with complex manipulations of an underlying database. In our case, the database is composed of relational tables encoding NCBI and ITIS taxonomies and a version of TreeBASE curated as detailed above. The database is handled by the MySQL database management system [13]. Although a Django project can consist of several applications, PhyloExplorer has currently only one application called "DjangoPhyloCore". "PhyloCore" is a wrapper of this library that enables users to access it outside any Django project, i.e. to use its facilities under the Python interpreter as if it were a standard library.

**Figure 1:**
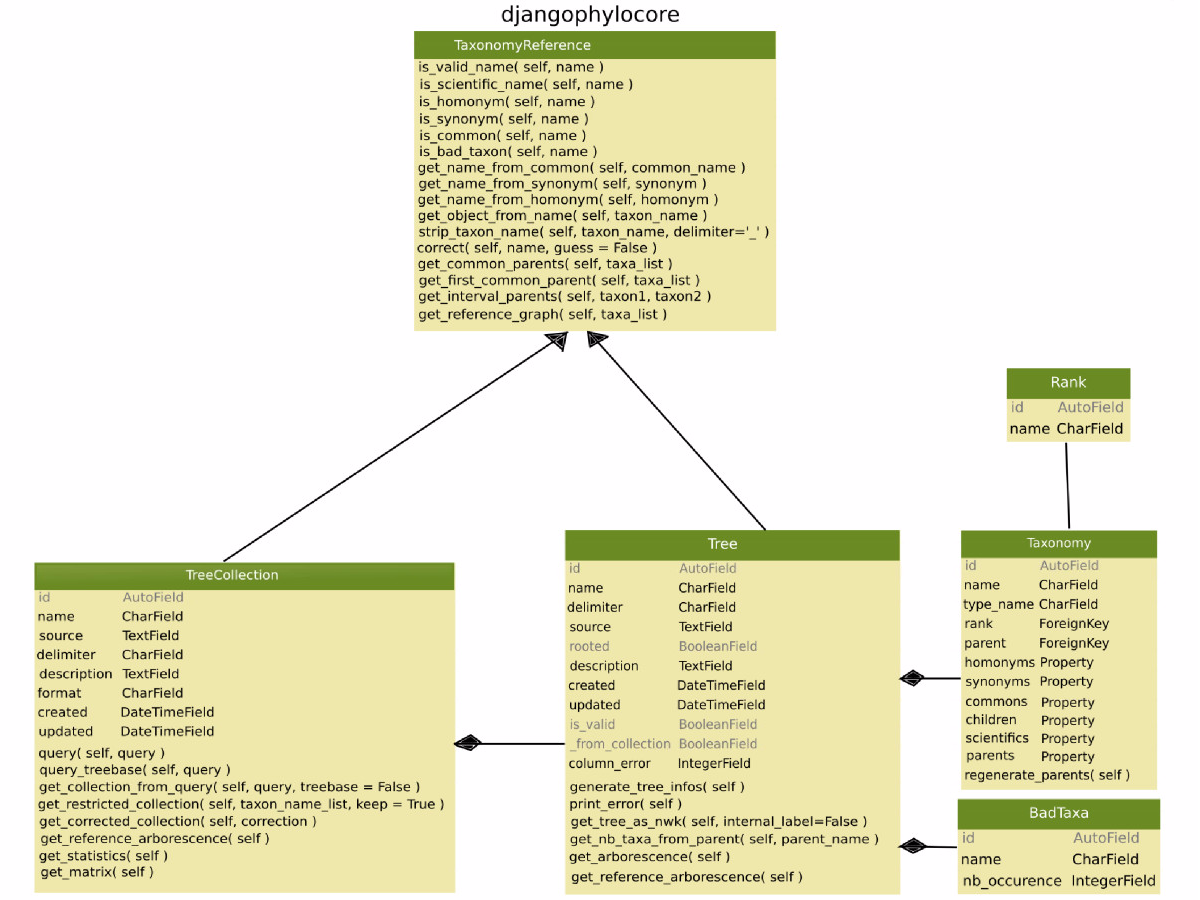
**UML diagram of the PhyloCore library structure of PhyloExplorer**.

The UML diagram structure of the "PhyloCore" library (Fig. 1) is composed of several classes. Most of them allow interaction with the corresponding tables in the database since Django imposes a class for each table of the database. The class "Tree" is used to store phylogenies as individual objects, including the Newick format and other associated properties such as a name or the tree's rooting status.

"TreeCollection" is the main class of the project and allows storage of user tree collections, TreeBASE itself, and sets of trees extracted from TreeBASE through the querying system. The queries are performed through functions of this class. The "TreeCollection" class also provides functions to filter trees by pruning them (e.g. restricting them to a taxonomic group of interest) and to obtain statistics on taxon coverage among trees. The "Taxonomy" class references all taxa of the current reference taxonomy (NCBI or ITIS) as well as their synonyms, homonyms, and hierarchical relationships between higher-level taxa. The "Rank" class encodes the taxonomic rank of the taxa (kingdom, phylum, class, family, genus, species), exactly as in the ITIS database [11]. The "TaxonomyReference" class provides useful functions common to trees and tree collection querying facilities. Lastly, the "BadTaxa" class stores taxa from currently managed tree collections that are not found in the chosen reference taxonomy.

The "PhyloCore" facilities can be accessed through a graphical interface provided by the PhyloExplorer web server. This server allows several users to simultaneously query "PhyloCore" and its database. The web server was developed using the CherryPy object-oriented HTTP framework for Python [14]. The NetworkX Python package [15] enables implementation of the browsable tree structures. PhyloExplorer relies on the PHY.FI graphical tool [16] for displaying labeled trees and also links to the PhyloWidget applet [17] for a less static tree display. Taxon pictures are extracted from the corresponding Wikispecies [18] and Wikipedia [19] pages. The PhyloExplorer source code is distributed under the CeCILL license version 3 [20], a French variant of the GNU GPL licence adopted by the Centre National de la Recherche Scientifique (CNRS). It can be downloaded from the following Google Code project page: .

## Results and Discussion

### Existing tools: software and websites

Many software packages propose to display and handle single user trees or collections of trees such as the long-standing classic Treeview [21], the sophisticated TreeDyn [22] and the rising star Dendroscope [23], among others. Websites aiming at interactively displaying phylogenetic trees, such as PHY.FI [16], iTOL [24] and PhyloWidget [17], for example, have also recently flourished. However, most of these tools are tree rendering programs that only allow the user to graphically manage trees and associated annotations in some cases. Database orientated web servers such as TreeFam [6] and PhyloFinder [25] permit taxonomic querying and retrieval of phylogenetic trees from dedicated databases. Whereas others, like GRUNT [26] and Summary Tree Explorer [27], implement some tree filtering and pruning features according to the taxonomy. However, none of these latter tools can be used to upload and explore user phylogenies or obtain detailed summary statistics on user tree collections. The only programs providing basic statistics on data availability are Clann [28] for tree collections in the context of supertree reconstruction, and PhyloTA Browser [29] for molecular datasets. In fact, PhyloFinder [25] is the closest conceptually to the PhyloExplorer tool presented here, but it is currently restricted to trees stored in TreeBASE and does not allow obtaining taxonomic coverage statistics or performing complex taxonomic queries.

### A brief overview of PhyloExplorer

A typical use of PhyloExplorer begins by uploading a tree collection on the website in Newick or Nexus format. A simple taxon list can also be entered as a trivial multifurcated tree. This allows users to deal with various kind of data (e.g. sequence alignments, morphological measures), for which the taxonomic representation is important. The trees are then parsed and taxon labels are mapped against scientific names from the chosen reference taxonomy (either NCBI or ITIS) with listed homonyms and synonyms. For automatic correction, the user is provided with a list of alternatives for up to ten unrecognized names. The corresponding excerpt of the reference taxonomy, including only mapped taxa, is then displayed. Two statistics are provided at each taxonomic rank. The first states the number of user trees containing a representative of the corresponding taxonomic group. The second states the number of representatives of this taxonomic group encountered in the user tree collection. Each mapped taxon is provided with a link to the corresponding entry in the NCBI or ITIS database. For pedagogical purposes, available pictures from Wikispecies [18] or Wikipedia [19] are popped up when browsing the taxonomic excerpt. Links to the corresponding iSpecies [30] and Wikispecies [18] pages are also provided for each taxon. A taxa × trees matrix scoring the presence or absence of taxa in trees of the user collection can be generated to visualize the taxonomic overlap among trees. The user can then perform several operations on the input collection, such as: browsing the taxonomy excerpt corresponding to each input tree; restricting its trees to a subset of species by a simple mouse click; mining a relevant subset of trees through a simple querying language; and locating trees in TreeBASE matching taxa of the input collection.

### Mapping and correcting taxon names

When PhyloExplorer is provided with a tree collection, either by simple upload or by searching TreeBASE, every leaf label is mapped to terms contained in the reference taxonomy. These terms include scientific and common names with their official taxonomic synonyms and homonyms. The lack of a single universal reference taxonomy [1] led us to consider several existing alternatives. By relying on the comprehensive, widely used, and up to date NCBI and ITIS taxonomic projects, we hope to be able to fulfil a broad spectrum of evolutionary biologists' needs. The PhyloExplorer taxon naming facility helps to detect misspelled or taxonomically incorrect taxon names and proposes corrections according to known synonymies. Leaf labels that cannot be mapped to the reference taxonomy are listed, as well as those having an ambiguous taxonomic name. As automatic correction is error prone, the user is prompted to correct problematic taxon names by selecting the appropriate name among close alternatives present in the reference taxonomy. Then PhyloExplorer will automatically correct taxon names accordingly in the whole tree collection. Corrected trees can be downloaded afterwards, as well as the detailed list of corrections.

**Figure 2:**
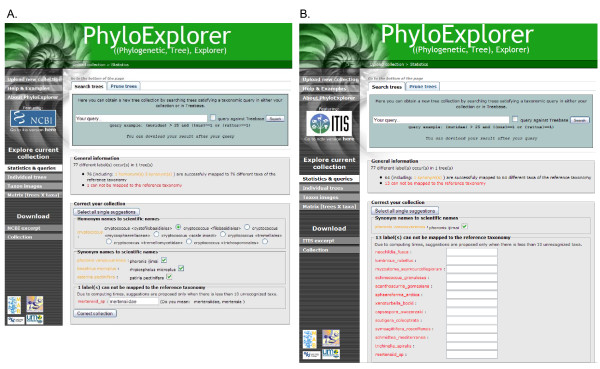
**Snapshots of PhyloExplorer's name correcting feature**. The 77 taxa metazoan phylogenetic tree obtained by Dunn *et al*. [31] [see Additional file 1] is checked against the NCBI (A) and ITIS (B) reference taxonomies. Note the many differences in name mapping induced by the use of a different taxonomic scheme.

**Figure 3:**
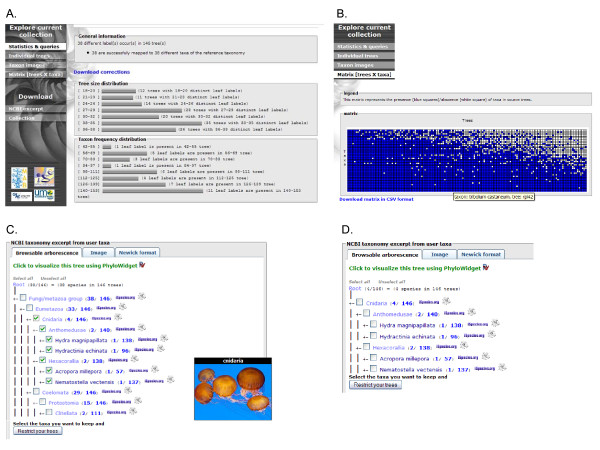
**Snapshots of PhyloExplorer's tree collection description and restriction features**. The 146-individual gene tree collection from the phylogenomic study of Delsuc *et al*. [32] [see Additional file 2] has been uploaded. A) PhyloExplorer produces statistics depicting the user tree collection in the form of simple tree size and taxon distribution histograms. B) Data availability matrix scoring the presence/absence of taxa in trees from the collection. C) Excerpt of the reference taxonomy (here NCBI) containing all mapped taxa from the tree collection with associated statistics at each node. For each taxonomic group, the number of its representatives found within the tree collection and for each taxon the number of user trees where it is represented. Here, all members of Cnidaria are selected from checkboxes to restrict the trees to members of this phylum. An illustrative picture is automatically popped up from the Wikispecies page for Cnidaria. D) Summary NCBI excerpt of the tree collection restricted to Cnidaria with adjusted statistics.

For example, we checked the 77-taxa metazoan phylogenetic tree from the recent study of Dunn *et al*. [31] (Fig. 2, Additional file 1). Using the NCBI reference taxonomy resulted in successful mapping of 76 leaf labels, including one homonym and three synonyms (Fig. 3A). Only one label (mertensiid_sp) could not be found in the reference taxonomy because it represents an English common name for an unidentified member of the family Mertensiidae. PhyloExplorer correctly suggested this name correction (Fig. 3A). By contrast, when using ITIS as the reference taxonomy, only one synonym and 13 labels that could not be mapped were revealed (Fig. 3B). This illustrates the differences in name mapping resulting from different taxonomic schemes. We hope that semi-automating this fastidious, but nevertheless essential task will encourage researchers to check the validity of their trees before depositing them in phylogenetic tree databases such as TreeBASE. This tree proofing stage is much easier with PhyloExplorer than with any other existing tool.

### Describing, browsing and restricting tree collections

As an illustration, we considered the collection of 146 individual gene trees from the phylogenomic study of Delsuc *et al*. [32](Additional file 2). This example is of particular interest because its trees contain both binomial species names and higher taxonomic rank names. The latter labels designate chimerical taxa assembled from gene sequences of different representatives, as is often the case in supermatrix studies [33]. Once this collection has been uploaded, PhyloExplorer produces simple statistics to depict it within the "Statistics & queries" view. Both tree size and taxon frequency distributions are summarized through graphical plots (Fig. 3A). The general information states that this dataset consists of a total of 146 trees containing 38 distinct taxa. The tree size distribution indicates that the smallest tree contains 18 taxa. The taxon frequency distribution reflects the substantial overlap among these trees, with no taxon appearing in less than 42 trees among 146 and most taxa occurring in more than a hundred different trees. When selecting the "Matrix trees × taxa" view, a data availability matrix is constructed from the collection (Fig. 3B). This representation provides a general overview of the degree of overlap among trees from the collection in the form of a matrix scoring the presence (blue squares) or absence (white squares) of each taxon in each tree. This matrix can easily be browsed by pointing the mouse on each matrix cell to display its associated taxon and tree name information. The detailed data availability matrix can also be downloaded in csv format (compatible with most spreadsheet programs) for use as supplementary material in supertree and supermatrix studies, for example.

By coming back to the "Statistics & queries" view, a summary excerpt of the reference taxonomy containing all mapped taxa is provided as an interactive tree (Fig. 3C). For educational purposes and to facilitate browsing of the taxonomy, images from Wikispecies [18] or Wikipedia [19] are automatically popped up when passing the mouse pointer over each taxonomic rank and taxon. The summary excerpt indicates, for each taxonomic rank, the number of its representatives contained in the tree collection and the number of user trees in which members of this taxon are represented. Based on these indications, it can easily be seen that four different cnidarians are represented in the whole tree collection and that 146 trees contain at least one cnidarian. A simple click on the first number allows the user to prune trees so that they only contain cnidarians, while clicking on the second will restrict the collection to the subset of trees containing at least one cnidarian. This latter function allows the user to easily restricting the tree collection to subsets of trees containing members of a particular taxonomic group or a terminal taxon of interest. PhyloExplorer also enables more complex and flexible tree restrictions by selecting relevant taxa through the corresponding checkboxes. Once the internal nodes of interest are selected, PhyloExplorer restricts the trees of the current collection to these taxa (Fig. 3D). The summary statistics are updated accordingly, and the modified collection can then be browsed through the taxonomic excerpt and downloaded in Newick format.

**Figure 4:**
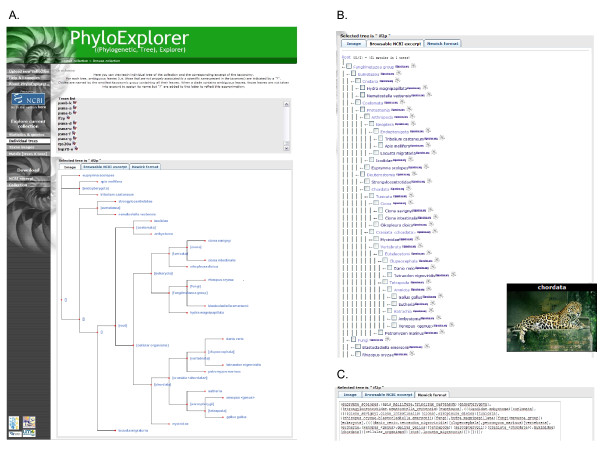
**Snapshots of PhyloExplorer's individual tree browsing and representation features**. A) Browsable tree list of 146 individual gene tree collection of Delsuc *et al*. [32] with links to PhyloWidget [17]. The 21-taxon tree inferred from the gene *if2p *is displayed using PHY.FI [16] with internal nodes labelled according to the NCBI reference taxonomy. B) Browsable NCBI excerpt corresponding to the *if2p *gene tree. C) Labelled *if2p *gene tree in Newick format.

The "Individual trees" view offers the option of browsing trees from the collection individually. A list of all individual trees is given with links for displaying each tree using the PhyloWidget [17] applet (Fig. 4A). Provided that trees have been named in the Nexus formatted collection, individual trees are listed after this name. Once a particular tree is selected, i.e. the tree from the *if2p *gene in the current example, its internal nodes are automatically labelled by PhyloExplorer with taxonomic ranks when they can be unambiguously inferred. The resulting decorated tree is rendered using PHY.FI [16] within the "Image" tab. The "Browsable taxonomy excerpt" tab allows the user to browse the tree in the reference taxonomy context with taxon image pop-ups and links to the iSpecies [30] and Wikispecies [18] pages of each taxon (Fig. 4B). Finally, the "Newick format" tab provides access to the labelled tree in Newick format (Fig. 4C).

### Performing complex taxonomic queries on tree collections

Finding a particular phylogeny in phylogenetic tree databases, or more generally in tree collections, is often like trying to find a needle in a haystack. Indeed, though confronted with phylogenetic tree databases containing thousands of trees, the user is usually offered only basic query capabilities. Recent efforts have been made in the right direction in the particular case of TreeBASE with tools designed to taxonomically validate the trees (TBMap) [3] and provide enhanced query options (PhyloFinder) [25]. However, a general system allowing complex taxonomic queries to be performed on taxonomically validated tree collections is still lacking. PhyloExplorer now offers this possibility.

**Figure 5:**
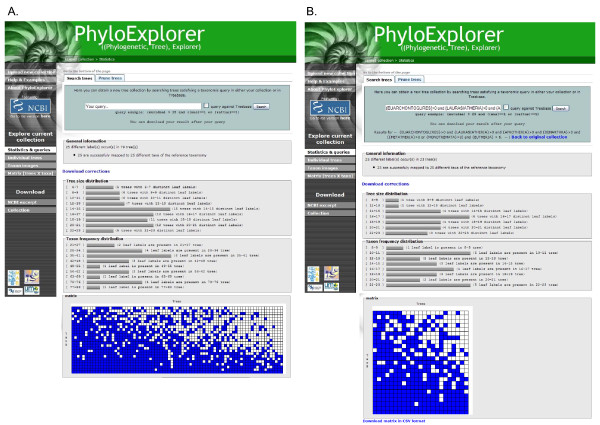
**Snapshots of PhyloExplorer's statistics and queries feature**. A) Statistics and data availability matrix for a mammalian tree collection containing 79 trees extracted from the OrthoMaM [7] [see Additional file 3]. B) Updated statistics and data availability matrix for the 23 trees filtered from the initial collection using the query: {EUARCHONTOGLIRES}>0 and {LAURASIATHERIA}>0 and {AFROTHERIA}>0 and {XENARTHRA}>0 and ({METATHERIA}>0 or {MONOTREMATA}>0) and {EUTHERIA} > 6.

For instance, we extracted a tree collection from the OrthoMam database [7] by performing a request aimed at selecting trees inferred from slow evolving markers (rate ≤ 0.5) of reasonable size (length > 1000 nucleotide sites). This led to a collection of 79 trees which was uploaded into PhyloExplorer (Fig. 5A, Additional file 3). This tree collection contains a total of 25 mammalian taxa for which complete genome sequences are available in the EnsEMBL database [8]. A question of primary interest in mammalian phylogenetics is the order in which diversification among the four major placental groups (Afrotheria, Xenarthra, Euarchontoglires and Laurasiatheria) occurred – a problem that is directly dependent upon the position of the placental root [34]. When studying the position of the root of the placental tree, it is better to consider trees or molecular datasets containing at least six eutherians and one representative of each major placental clade, plus a marsupial or monotreme outgroup. Such trees can be located very easily in PhyloExplorer by applying the following query to the OrthoMam trees under the "Search trees" tab: {EUARCHONTOGLIRES}>0 and {LAURASIATHERIA}>0 and {AFROTHERIA}>0 and {XENARTHRA}>0 and ({METATHERIA}>0 or {MONOTREMATA}>0) and {EUTHERIA} > 6. For each tree T of the current collection, PhyloExplorer replaces {TAXONOMIC_GROUP} by the number of representatives of this taxonomic group within T. Tree T matches the query if the resulting Boolean expression is true. The above query returned 23 candidate trees, while providing an even sampling of the major placental clades (Fig. 5B). Then, the user can conduct a supertree analysis from the indicated subcollection with an external tool. Alternatively, the user can collect the corresponding datasets in the OrthoMam database to construct a supermatrix with an optimal distribution of missing data with regards to the biological question at issue.

PhyloExplorer's query language can also be used to mine trees from the TBMap-curated version of TreeBASE. The same request for mammalian trees on TreeBASE results in 13 trees containing a total of 210 distinct taxa from which a supertree can be reconstructed. This querying of TreeBASE is also helpful for supermatrix studies, as primary data matrices can be downloaded from the TreeBASE website once relevant study numbers have been identified thanks to PhyloExplorer. Finally, the "Pruning trees" tab provides an easy way to restrict tree collections by defining taxonomic filters. Once filters have been defined, trees can then be restricted to include only taxa respecting these filters, or alternatively be pruned from the taxa defined in these filters. This function can be particularly useful for testing the influence of taxon sampling on diversification studies which require handling and statistical analysis of very large trees [35].

### Measuring the taxonomic content and coverage of TreeBASE

**Figure 6:**
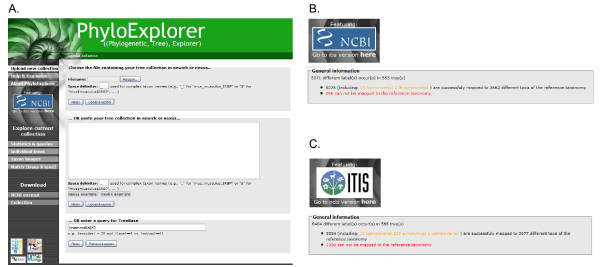
**Snapshots of PhyloExplorer's TreeBase statistics and query features**. A) PhyloExplorer home page showing the simple query {mammalia}>0 that allows users to retrieved all trees containing at least one mammalian taxon from our TBMap-curated [3] version of TreeBASE [2]. B) Taxonomic mapping of the resulting TreeBASE collection using the NCBI reference taxonomy. C) Taxonomic mapping of the resulting TreeBASE collection using the ITIS reference taxonomy.

The TBMap-curated version of TreeBASE [2] we use includes 6,237 trees. However, its taxonomic coverage is highly irregular, with some groups being much more represented than others. Moreover, islands of taxa exist in which constitutive taxa are not linked by any tree to other taxa. This has, in particular, been pointed out by Page [3], who provided several graphical views of the situation as of 2004. However, a more detailed and live picture can be obtained as TreeBASE is available as a specific collection in PhyloExplorer. The complete TreeBASE tree collection can be queried taxonomically from the PhyloExplorer front page (Fig. 6A). This enables the user to query and investigate its taxonomic coverage much more closely and dynamically than is possible when using the TreeBASE website interface or even the PhyloFinder dedicated tool [25]. Note, however, that some taxa appearing in TreeBASE cannot be mapped to our reference taxonomies despite the use of the TBMap-curated version of TreeBASE [3]. Also, the query result depends on the chosen reference taxonomy. For example, the simple query {mammalian}>0, allows retrieving all trees from TreeBASE that contain at least one mammal (Fig. 6A). Using the NCBI reference taxonomy results in 553 trees containing 5,371 different labels of which 5,075 (including 23 homonyms 176 synonyms) are successfully mapped to 3,660 distinct taxa (Fig. 6B). Whereas, using the ITIS reference taxonomy returns 566 trees containing 6,404 different labels of which 5,054 (including 10 homonyms, 127 synonyms and 1 vernacular) are successfully mapped to 3,677 distinct taxa (Fig. 6C). We plan to annually update the TreeBASE content of our site.

### Educational value of the web server

PhyloExplorer also provides a useful educational tool for assessing a taxonomic group that the user is not familiar with. This feature could be of particular value when preparing practical courses for undergraduates. Indeed, when uploading a tree or a list of taxa of interest, PhyloExplorer constructs an excerpted version of the corresponding taxonomic tree that can be browsed interactively. Internal nodes of the taxonomic excerpt tree are labelled by the name of the smallest taxonomic rank containing all taxa belonging to this group. For example, PhyloExplorer can be used to quickly view and browse the taxonomy of taxa represented in the NCBI Trace Archives [36]. Pictures of taxa available in Wikispecies [18] can be popped-up by positioning the mouse pointer over the corresponding taxon name. Clicking on taxon names will redirect the user to the full taxon description in the NCBI Taxonomy Browser [37], which indicates the number of nucleotide sequences available for that particular taxon. PhyloExplorer also provides a link to automatically perform a search for each taxon in the iSpecies engine [30], which returns information on the geographic distribution, available pictures on the web, and associated bibliography. A link is also given for each taxon to the corresponding Wikispecies page for additional information.

**Figure 7:**
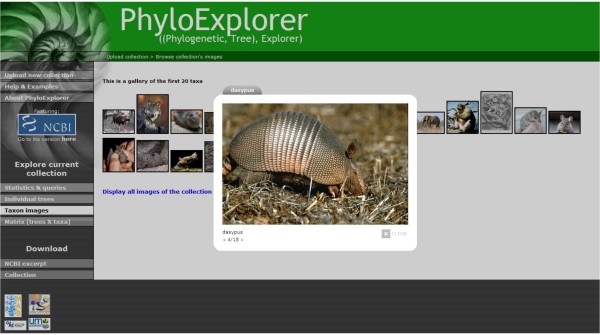
**Snapshot of PhyloExplorer's image search feature**. Taxon images are searched for the 20 first taxa of the OrthoMaM-based tree collection [see Additional file 3]. The 18 images found are displayed as thumbnails and the full-size picture of the armadillo (*Dasypus*) is viewed within a slideshow where all available images can be browsed.

Taxon pictures are of great educational value in many cases in which a phylogeny is to be shown or described. It is indeed common practice to display such taxon images at the tips of phylogenies in slide presentations, posters, and publications. Such pictures can be found on dedicated websites such as Wikispecies or Animal Diversity Web [38], or through search engines such as iSpecies. However, manually typing each taxon name in these websites can be cumbersome and is prone to spelling errors. PhyloExplorer automates this task by providing a feature to get available pictures from Wikispecies for all terminal taxa of an input tree or tree collection and for all taxa of a taxon list. Shifting to the "Taxon images" view prompts PhyloExplorer to display, by default, available images for the first 20 taxa. Annotated thumbnails of retrieved images are returned and the collection of full-size images can be viewed as a slide show (Fig. 7). A link is also provided to launch the search for all taxon images in a separate window of the browser. The pictures can then be downloaded for direct use in scientific presentations and lectures. An interactive tree viewer program like Dendroscope [23] can also directly upload these pictures for display at the tips of trees by mapping taxon names with image filenames.

## Conclusion

Combining user phylogenies with a reference taxonomy allows PhyloExplorer to propose advanced facilities to taxonomically explore, correct, query and filter user tree collections. Various cumbersome operations currently performed by hand can thus be automated – in the best case – before publishing phylogenies in papers or repositories. PhyloExplorer's features are available through a simple interactive web interface. In addition, the taxonomic querying system can also be applied to a TBMap-curated version of TreeBASE [3] which can thus be efficiently mined with respect to an input tree collection or to the user's interests. We also hope that PhyloExplorer will benefit the phylogenetic community as a tool to increase the taxonomic validity of trees submitted to phylogenetic tree databases. This would greatly enhance the usefulness of such databases in meta-analyses. Moreover, by providing a powerful taxonomic query system that enables the mining of user collections or databases containing hundreds to thousands of trees, PhyloExplorer could eventually be of great assistance to researchers interested in performing large-scale supertree and supermatrix analyses. PhyloExplorer's educational potential should also be underlined as it might be particularly helpful for preparing undergraduate courses and boosting public awarenes on taxonomy and phylogenetics. Possible developments of PhyloExplorer include allowing users to upload their own custom reference taxonomic schemes, downloading standardised taxon image collections, adding more tree handling operations such as automatic re-rooting, and implementing topological queries.

## Availability and requirements

Project name: PhyloExplorer

Project home page: 

Code available at: 

Operating system(s): Platform independent

Other requirements: None.

Licence: CeCILL v3.

## Authors' contributions

VR initiated the project. FD, VB, and VR supervised the project. NA, NC and SD, three Master's students at University Montpellier II contributed to the implementation of the Python library and the development of the first website version. NC and VR wrote an updated version of the website. SP, a Master's student at University Montpellier II, extracted the trees from TreeBASE and converted them according to the TBMap and Catalogue of Life resources. FD provided the illustrative biological examples. FD, VB and VR wrote the manuscript. All authors read and approved the final manuscript.

## Supplementary Material

Additional file 1 **77-taxon metazoan phylogenetic tree**. This file contains the metazoan phylogenetic tree obtained by Dunn *et al*. [31] in nexus format. Click here for file

Additional file 2 **Collection of 146 phylogenetic trees**. This file contains the tree collection (146 trees) from the phylogenomic study of Delsuc *et al*. [32] in nexus format. Click here for file

Additional file 3 **Collection of 79 mammalian phylogenetic trees**. This file contains the mammalian tree collection (79 trees) extracted from the OrthoMam database [7] in nexus format. Click here for file
